# Association between anthropometric indicators of adiposity and hypertension in a Brazilian population: Baependi Heart Study

**DOI:** 10.1371/journal.pone.0185225

**Published:** 2017-10-12

**Authors:** Camila Maciel de Oliveira, Anderson Zampier Ulbrich, Felipe Silva Neves, Fernando Augusto Lavezzo Dias, Andréa Roseli Vançan Russo Horimoto, José Eduardo Krieger, Rafael de Oliveira Alvim, Alexandre da Costa Pereira

**Affiliations:** 1 Laboratory of Genetics and Molecular Cardiology, Heart Institute (InCor) do Hospital das Clínicas da Faculdade de Medicina da Universidade de São Paulo, São Paulo, SP, Brazil; 2 Department of Integrative Medicine, Center for Health Sciences, Federal University of Paraná (UFPR), Curitiba, PR, Brazil; 3 Department of Nutrition, Institute of Biological Sciences, Federal University of Juiz de Fora (UFJF), Juiz de Fora, MG, Brazil; 4 Department of Physiology, Federal University of Paraná (UFPR), Curitiba, PR, Brazil; 5 Postgraduate Program in Public Health, Federal University of Espírito Santo (UFES), Vitória, ES, Brazil; The University of Tokyo, JAPAN

## Abstract

**Background:**

Recently, some studies have evaluated the role of adiposity measures in the prediction of hypertension risk, but the results are conflicting. Thus, the aim of this study was to compare which of the four indicators of adiposity (waist circumference–WC, body mass index–BMI, body adiposity index–BAI, and visceral adiposity index–VAI) were better associated with hypertension in a Brazilian population.

**Methods and findings:**

For this study, were selected 1627 individuals (both genders, and aged over 18 years) resident in the municipality of Baependi, a city located in the Southeast of Brazil. WC, BMI, BAI and VAI were determined according to a standard protocol. Hypertension was defined as mean systolic blood pressure ≥ 140 mmHg and/or diastolic blood pressure ≥ 90 mmHg, and/or antihypertensive drug use. The indicators of adiposity WC, BMI, BAI, and VAI were higher in hypertensive when compared to non-hypertensive individuals. In addition, WC and BMI were most strongly associated with hypertension in men and women, respectively. The area under the curve (AUC) of WC was significantly higher than VAI in men. In women, both AUC of BMI and WC showed higher discriminatory power to predict hypertension than BAI and VAI.

**Conclusions:**

The indicators of adiposity WC and BMI were better associated with hypertension than BAI and VAI, in both genders, and it could be a useful tools for the screening of hypertensive patients.

## Introduction

Hypertension is a multifactorial clinical condition characterized by high and sustained levels of blood pressure (BP) [1]. In the last years, epidemiological studies have shown that higher levels of BP are widely associated with increased risk of fatal and nonfatal cardiovascular events [2–5]. Hypertension is a highly prevalent disease, affects about 1.13 billion adults worldwide [6], and is associated with several cardiometabolic conditions such as hyperlipidemia, atherosclerosis, inflammation, insulin resistance, diabetes mellitus, and obesity [4,5,7–10].

The strong association between obesity and cardiometabolic disorders motivated the development of several techniques simple and low-cost used to determine body adiposity [11,12], such as body mass index (BMI), waist circumference (WC) and body adiposity index (BAI) [13–15]. Recently, Amato et al. [16] suggested visceral adiposity index (VAI) to predict cardiometabolic risk in adult women and men. Furthermore, Ding et al. [17] demonstrated a significant relationship between BP levels and VAI, in a Chinese population. However, it is still needed further investigation that allows clarifying the advantages of this tool in the screening of hypertensive patients.

Thus, the aim of this study was to compare which of the four indicators of adiposity (VAI, WC, BMI, and BAI) were better associated with hypertension in a Brazilian population.

## Methods

### Study population

The *Baependi Heart Study* is a genetic epidemiological study of cardiovascular disease risk factors [18]. For this study, we carried out a cross-sectional analysis of data collected between December 2010 and January 2013. One hundred and nine families (1,627 individuals of both genders and aged 18–102 years) were selected in Baependi, a city in a rural area (752 Km^2^, 18,307 inhabitants) located in Minas Gerais State, Brazil. Probands were identified from the community at large in several stages. First, eleven census districts (from a total of twelve) were selected for study. Second, residential addresses within each district were randomly selected (first by randomly selecting a street, second a household). Finally, eligibility criteria (any individual living in the selected household who was 18 years old or above) within each household were established. Once a proband was enrolled, all his/her first-degree (eg, parents, siblings, and offspring), second-degree (eg, halfsiblings, grandparents/grandchildren, aunts/uncles, nieces/nephews, and double cousins), and third-degree (eg, first cousins, great-uncles/great-aunts, and greatnephews/great-nieces) relatives and his/her respective spouse's relatives, who were at least 18 years old, were invited to participate. After the proband's first contact, first degree relatives were invited to participate by phone; these included all living relatives in the city of Baependi (urban and rural area) and surrounding cities [18].

To recruit the participants, the project was advertised through provincial, religious, and municipal authorities, in local television, newspaper, and radio messages, through physicians, and by phone calls. For physical examination, a clinic was established in a quiet but easily accessible sector of Baependi. Only individuals age 18 and older were eligible to participate in the study [18].

The study protocol was approved by the ethics committee of the *Hospital das Clínicas* (SDC: 3485/10/074), University of São Paulo, Brazil, and each subject provided informed written consent before participation.

### Blood pressure measurements

BP was measured using a standard digital sphygmomanometer (OMRON, Brazil) on the left arm after 5 min rest, in the sitting position. Systolic (SBP) and diastolic blood pressures (DBP) were calculated from three readings (mean value of all measurements), with a minimal interval of 3 min [18]. The mean BP (MBP) was calculated as DBP plus one-third pulse pressure. Hypertension was defined as mean SBP ≥ 140 mmHg and/or DBP ≥ 90 mmHg and/or antihypertensive drug use [19].

### Biochemical measurements

Blood triglycerides (TG), total cholesterol, high-density lipoprotein cholesterol (HDL-c), low-density lipoprotein cholesterol (LDL-c) and fasting glucose were evaluated by standard techniques in 12-h fasting blood samples [18]. Glycated hemoglobin (HbA1c) levels were determined by high-performance liquid chromatography (HPLC, National Glycohemoglobin Standardization Program, USA). Diabetes mellitus was diagnosed by the presence of fasting glucose ≥ 126 mg/dL, HbA1c ≥ 6.5%, or antidiabetic drug use.

### Anthropometrical indicators of adiposity measurements

Anthropometric parameters were measured according to a standard protocol [18]. Height was measured in centimeters and weight in kilograms using a calibrated digital balance. WC was measured at the mean point between the lowest rib margin and the iliac crest with the subject standing and at the maximum point of normal expiration. Hip circumference was measured to the nearest 0.1 cm around the thighs, at the height of the greater trochanter, in the standing position.

BMI was calculated as body weight (Kg) divided by height squared (m^2^) and obesity defined as BMI ≥ 30 Kg/m^2^.

BAI was calculated using hip circumference and height [15]:

$$\mathrm{BAI}=\left\{\left[\mathrm{hip}\;\left(\mathrm{cm}\right)/\mathrm{height}\;{\left(m\right)}^{1.5}\right]\;\text{-}\;18\right\}$$

VAI, a sex-specific index based on WC, BMI, TG and HDL-c, was calculated as follows [16]:

Males:VAI={WC/[39.68+(1.88*BMI)]}*(TG/1.03)*(1.31/HDL-c)

Females:VAI={WC/[39.58+(1.89*BMI)]}*(TG/0.81)*(1.52/HDL-c)

### Statistics analyses

Categorical variables are presented as percentages, whereas continuous variables are presented as mean ± standard deviation. To evaluate the performance models, Receiver Operational Characteristic (ROC) curves were built and the area under the curve (AUC) was used to measure the discriminatory power for hypertension in men and women. AUC the ROC curves between the markers were compared using a parametric method, with GraphROC for Windows software [20]. Sensitivity and specificity values for each measure of adiposity were determined by ROC curves analysis. The optimal cutoff points for WC, BMI, BAI and VAI were established based on the highest combination of sensitivity and specificity. Logistic regression analyses were used to assess the association between the different measures of adiposity (WC, BMI, BAI and VAI) and hypertension. All analyses were adjusted by age, HbA1c, LDL-c, HDL-c and triglycerides. These confounding variables have been chosen on the basis of biological plausibility. Statistical analyses were carried out using SPSS software (version 19.0, Chicago, IL, USA), with the level of significance set at 5%.

## Results

Demographic data related to age, WC, BMI, BAI, VAI, BP phenotypes, lipid profile, fasting glucose, HbA1c, and presence of diabetes and obesity, stratified by hypertension status in the whole sample are summarized in Table 1.

**Table 1:** General characteristics of subjects stratified by hypertension.

Characteristics	Non-Hypertensive	Hypertensive	p
n	976	651	*****
Age (years)	38.3 ± 13.9	54.8 ± 14.7	< 0.001
Gender, male (%)	41.8	41.0	0.400
Obesity (%)	10.7	31.8	< 0.001
WC (cm)	87.9 ± 10.5	96.2 ± 12.5	< 0.001
BMI (Kg/m^2^)	24.5 ± 4.2	27.7 ± 5.7	< 0.001
BAI	28.1 ± 5.4	31.0 ± 6.6	< 0.001
VAI	1.9 ± 1.2	2.6 ± 1.8	< 0.001
SBP (mmHg)	117.6 ± 10.3	137.2 ± 16.5	< 0.001
DBP (mmHg)	72.4 ± 7.9	81.9 ± 11.1	< 0.001
MBP (mmHg)	87.5 ± 7.9	100.3 ± 11.4	< 0.001
Diabetes (%)	2.9	15.1	< 0.001
Fasting glucose (mg/dL)	88.6 ± 14.0	98.4 ± 23.5	< 0.001
HbA1c (%)	5.5 ± 0.6	5.9 ± 0.9	< 0.001
Total cholesterol (mg/dL)	192.1 ± 39.6	206.4 ± 40.8	< 0.001
LDL-c (mg/dL)	121.1 ± 34.8	129.8 ± 35.8	< 0.001
HDL-c (mg/dL)	47.7 ± 11.6	46.4 ± 12.1	0.030
Triglycerides (mg/dL)	116.4 ± 54.3	151.0 ± 80.2	< 0.001

WC, waist circumference; BMI, body mass index; BAI, body adiposity index; VAI, visceral adiposity index; SBP, systolic blood pressure; DBP, diastolic blood pressure; MBP, mean blood pressure; HbA1c, glycated hemoglobin; LDL-c, low density lipoprotein; HDL-c, high density lipoprotein.

Diabetes: fasting glucose ≥ 126 mg/dL and/or use of hypoglycemic drugs.

Continuous data are expressed as mean ± standard deviation.

Categorical data are expressed as percentage.

**Fig 1:**
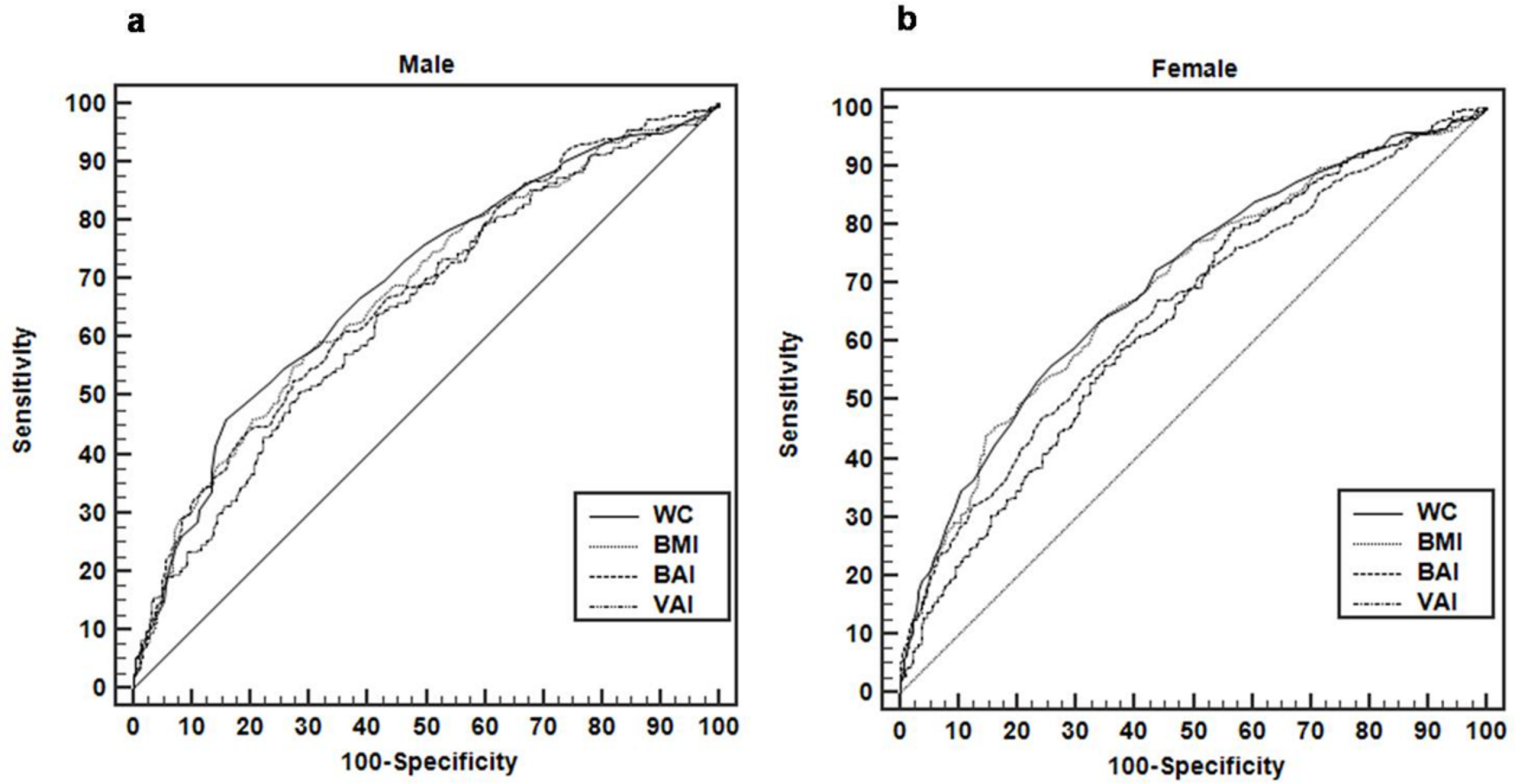
Discriminatory power of anthropometric indicators of adiposity in the hypertension screening in a Brazilian population. Area under the ROC curve (AUC): (a) male and (b) female. WC, waist circumference; BMI, body mass index; BAI, body adiposity index; VAI, visceral adiposity index. The AUC, cutoffs, sensitivity and specificity values are shown in Table 2.

Fig 1 contains graphical representations of ROC curves of anthropometric indicators of adiposity in the hypertension screening for male (Fig 1A) and female (Fig 1B). Cutoffs, sensitivity, specificity and AUC are reported in Table 2. In men, the discriminatory powers of WC, BMI and BAI in the prediction of hypertension were not different. However, the AUC of WC was significantly higher than that of VAI (0.69 vs 0.65, p = 0.030, respectively). In women, the AUC of WC was significantly higher than that of BAI (0.70 vs 0.65, p = 0.002, respectively) and VAI (0.70 vs 0.65, p = 0.004, respectively). In addition, the AUC of BMI also was significantly higher than that of BAI (0.69 vs 0.65, p < 0.001, respectively), and VAI (0.69 vs 0.65, p = 0.020, respectively). As expected, differences in the optimum cutoff values between men and women were observed. Sensitivity and specificity of the different indices were similar across indices and sexes, with the exception of a higher sensitivity observed for VAI, in women.

**Table 2:** Determination of the optimal cutoffs values and AUC for WC, BMI, BAI and VAI in a Brazilian population.

Gender	Anthropometric indicators of adiposity	Cutoffs	Sensitivity (%)	Specificity (%)	AUC (95% CI)	p
Male	WC	96.0	45.7	84.1	0.69 (0.65–0.72)*	< 0.001
	BMI	25.5	54.7	72.8	0.68 (0.64–0.71)	< 0.001
	BAI	25.8	52.6	72.7	0.67 (0.63–0.70)	< 0.001
	VAI	1.91	51.1	71.6	0.65 (0.61–0.68)	< 0.001
Female	WC	94.0	56.2	74.5	0.70 (0.67–0.73)*^†^	< 0.001
	BMI	28.7	45.3	84.0	0.69 (0.66–0.72)^‡^^¶^	< 0.001
	BAI	31.2	66.9	56.0	0.65 (0.62–0.68)	< 0.001
	VAI	1.56	79.9	43.1	0.65 (0.61–0.68)	< 0.001

WC, waist circumference; BMI, body mass index; BAI, body adiposity index; VAI, visceral adiposity index; AUC, area under the ROC curve; CI, confidence interval.

*WC vs VAI, p = 0.03.

^†^WC vs BAI, p = 0.002.

^‡^BMI vs BAI, p ≤ 0.001.

^¶^BMI vs VAI, p = 0.02.

The logistic regression analysis (Table 3) showed that, in men, WC (OR = 3.89 [95% CI, 2.55–5.95]) was better associated with hypertension than BMI (OR = 3.09 [95% CI, 2.08–4.59]), BAI (OR = 2.13 [95% CI, 1.46–3.12]), and VAI (OR = 1.40 [95% CI, 0.78–2.52]). However, in women, BMI (OR = 3.30 [95% CI, 2.31–4.73]) was better associated with hypertension than WC (OR = 2.19 [95% CI, 1.57–3.01]), BAI (OR = 1.82 [95% CI, 1.31–2.53]), and VAI (OR = 2.06 [95% CI, 1.26–3.37]). All analyses were adjusted for age, HbA1c, LDL-c, HDL-c and triglycerides.

**Table 3:** Association between anthropometric indicators of adiposity and hypertension by logistic regression multivariate analysis in a Brazilian population.

Anthropometric indicators of adiposity	Hypertension			
	Male		Female	
	OR (95% CI)	p	OR (95% CI)	p
WC	3.89 (2.55–5.95)	< 0.001	2.19 (1.57–3.01)	< 0.001
BMI	3.09 (2.08–4.59)	< 0.001	3.30 (2.31–4.73)	< 0.001
BAI	2.13 (1.46–3.12)	< 0.001	1.82 (1.31–2.53)	< 0.001
VAI	1.40 (0.78–2.52)	0.260	2.06 (1.26–3.37)	0.004

WC, waist circumference; BMI, body mass index; BAI, body adiposity index; VAI, visceral adiposity index; OR, odds ratio; CI, confidence interval.

BMI: cutoffs (men > 25.5 and women > 28.7).

WC: cutoffs (men > 96 and women > 94).

BAI: cutoffs (men > 25.8 and women > 31.2).

VAI: cutoffs (men > 1.91 and women > 1.56).

Hypertension: mean systolic blood pressure ≥ 140 mmHg and/or diastolic blood pressure ≥ 90 mmHg or use of anti-hypertension drugs.

All analyses were adjusted for age, HbA1c, LDL-c, HDL-c and triglycerides.

## Discussion

The main finding of our study is that WC, in men, and BMI, in women, were the anthropometric indicators of adiposity most strongly associated with hypertension. In addition, our data showed that the AUC of WC was significantly higher than VAI, in men. In women, both AUC of BMI and WC showed higher discriminatory power to predict hypertension than BAI, and VAI.

Recently, several studies have showed that overweight are one of the principal factors related to increased incidence of hypertension in worldwide [1,21–23]. Obesity individuals have some clinical features such as hypervolemia, increased peripheral resistance associated to high cardiac output, sympathetic hyperactivity, and inflammation [12,21,22]. Moreover, high percentage of fat, mainly visceral, have been related to insulin resistance, high levels of angiotensin II, increased secretion of aldosterone, and, consequently, high absorption of sodium in renal tubules [12,21–23].

As expected, in our study, hypertensive participants showed high values of WC, BMI, BAI, VAI, fasting glucose, HbA1c, total cholesterol, LDL-c, and triglycerides. However, we observed the opposite with HDL-c.

One of the most relevant risk factors related to hypertension is obesity, which is considered an important risk factor for type 2 diabetes mellitus as well. Nowadays, explaining this relationship has been a challenge in some fields of research and it involves insulin resistance mechanisms [21–23]. In this Brazilian population, obesity was more prevalent among hypertensive individuals (31.8% vs 10.7%). In addition, hypertensive individuals had higher values anthropometric measurement of adiposity such as WC, BMI, BAI and VAI. Besides that, it is expected the higher proportion of hypertension among diabetic individuals (15.1%) than normotensive groups (2.9%). These results are in agreement with the literature, and the relationship among obesity, hyperlipidemia, diabetes, and high levels of BP has been shown in several studies [5,6,8,9,11,12,17,21–23].

Of four measures of adiposity tested, the BMI and BAI are associated to total body fat. Though BMI is more used in the clinical practice to determine overweight, obesity, and cardiometabolic risk [13,14,24]. However, WC and VAI are indices better related to visceral fat [13,16,24].

In our study, we identified that both WC and BMI were more strongly associated to hypertension. In physically active individuals, it was observed that the elevation of 2.4 Kg/m^2^ in BMI is associated with an increase in hypertension risk [1]. Tuan et al. [12] studying 7336 Chineses, showed that the high prevalence of hypertension has been associated with increased WC, BMI, waist-to-height ratio, and waist-to-hip ratio. In the same study, BMI was the best predictor of hypertension. Contrasting these findings, Lee et al. [11], studying individuals in the *Korean Genome and Epidemiology Study*, related that hazard ratio and AUC of BMI were lower than that WC, in both genders. Knowles et al. [25], assessing 1,518 participants from Peru, demonstrated that WC was the best measure of body fat to predict high values of blood pressure, in men. In addition, data from meta-analysis involving 88,000 participants of several studies showed that BMI had the lowest discriminatory power to hypertension, in both genders [26].

In 2011, the BAI was suggested as an index highly accurate to measure the percentage of general body fat in women and men of several ethnicities [15]. Recent investigations showed the importance of this index in the assessment of body composition, and its relationship with cardiometabolic risk factors. Furthermore, its predictive role has been compared to other measures of adiposity such as WC, BMI, waist-to-height ratio, and waist-to-hip ratio [13,23,27]. However, studies demonstrating the discriminatory power of BAI in the prediction of hypertension are still scarce. In our study, we demonstrated that WC and BMI were superior to BAI, in women. Corroborating such results, Melmer et al. [28] evaluating 1,770 participants of *SAPHIR Study* showed that BAI was inferior than BMI, and waist-to-height ratio in the prediction of high levels of systolic and diastolic blood pressure. In the other hand, we observed that the discriminatory power of WC, BMI, and BAI were similar in men. Recently, D´Elia et al. [29], studying 350 health men after eight years follow-up, showed that both BAI and BMI were good predictors to hypertension and changes in BP levels.

Regarding the VAI, several studies have demonstrated its usefulness in measuring of visceral fat [23,30], prediction of metabolic syndrome [31] and arterial stiffness [32], and as risk marker to cardiometabolic diseases [25,30,33–35,36]. Elisha et al. [37], analyzing 99 overweight women in the postmenopausal period, observed that the VAI was a weak predictor to change of visceral fat percentage after a weight loss program for six months. Similarly to BAI, studies demonstrating the association of VAI with phenotypes related to hypertension are scarce. In our findings, VAI presented lower predictive power to hypertension when compared to other indices, in men. However, in women, the VAI was only superior to BAI. Differently, Ding et al. [17], studying 4065 Chinese, showed that VAI was related to BP in both genders, independently of confounding factors such as age, smoking, alcohol consumption, physical activity level, fasting glucose, plasmatic insulin, diabetes, and others. In the same study, the authors showed that the VAI was a good predictor to prehypertension.

Part of the controversial results present in this discussion may be explained by the following factors: First, the different prevalence of hypertension among several populations studied in the literature could affect the discriminatory power of measures of adiposity. Second, the distinct distribution of body fat among Eastern and Western, Asian, and Caucasian, generates a large variation in the predictive power of these measures of adiposity [24,25,38,39]. Third, the measures of adiposity are highly correlated (r > 0.8), and the resulting colinearity could lead to unstable associations for each of these variables [12,40]. Fourth, until this moment, none of the measures of adiposity investigated are strong enough to be used alone in the prediction of cardiometabolic risk factors. Fifth, the use of different statistic methods could contribute to controversial results.

Lastly, our study presents some advantages. We used a rigorous method, ensuring internal validity of the data collected. Moreover, regarding to hypertension, few studies have tested the predictive power of BAI and VAI. However, some limitations should also be considered. First, in our population, we didn’t compare measures of adiposity to evaluate body fat such as dual energy X ray absorptiometry (DXA). Second, because it is a cross-sectional study greater care is needed in inferring a cause, prognosis or a natural history of a disease. As a prevalence is the product of the incidence and duration of the disease, a factor that is associated with higher prevalence may be a cause of the disease, may also be associated with its prolonged duration [41]. Therefore, a causal relationship between body fat (as measured by adiposity measures) and hypertension could not be established.

In summary, WC and BMI were better associated with hypertension than BAI and VAI, in both genders. Thus, it is plausible to surmise that both, WC and BMI, can be useful tools in the screening of hypertensive patients belonging to a Brazilian population.

## Supporting information

S1 File Data set used to reach the conclusions. WC, waist circumference; BMI, body mass index; BAI, body adiposity index; VAI, visceral adiposity index; SBP, systolic blood pressure; DBP, diastolic blood pressure; MBP, mean blood pressure; LDL-c, low density lipoprotein; HDL-c, high density lipoprotein.
(XLSX)
